# Promotion of a healthy lifestyle among 5-year-old overweight children: health behavior outcomes of the 'Be active, eat right’ study

**DOI:** 10.1186/1471-2458-14-59

**Published:** 2014-01-21

**Authors:** Amy van Grieken, Carry M Renders, Lydian Veldhuis, Caspar WN Looman, Remy A Hirasing, Hein Raat

**Affiliations:** 1 Department of Public Health, Erasmus MC University Medical Center Rotterdam, P.O. Box 2040, Rotterdam 3000 CA, the Netherlands; 2 Department of Health Sciences, Faculty of Earth and Life Sciences, VU University Amsterdam, Amsterdam, The Netherlands; 3 EMGO Institute for Health and Care Research, VU University Amsterdam, Amsterdam, The Netherlands; 4 Department of Public and Occupational Health, VU University Medical Center, Amsterdam, The Netherlands

**Keywords:** Obesity, Overweight, Prevention, Intervention, Elementary school, Preschool, Children, RCT, Youth health care

## Abstract

**Background:**

This study evaluates the effects of an intervention performed by youth health care professionals on child health behaviors. The intervention consisted of offering healthy lifestyle counseling to parents of overweight (not obese) 5-year-old children. Effects of the intervention on the child having breakfast, drinking sweet beverages, watching television and playing outside were evaluated.

**Methods:**

Data were collected with the 'Be active, eat right’ study, a cluster randomized controlled trial among nine youth health care centers in the Netherlands. Parents of overweight children received lifestyle counseling according to the intervention protocol in the intervention condition (n = 349) and usual care in the control condition (n = 288). Parents completed questionnaires regarding demographic characteristics, health behaviors and the home environment at baseline and at 2-year follow-up. Cluster adjusted regression models were applied; interaction terms were explored.

**Results:**

The population for analysis consisted of 38.1% boys; mean age 5.8 [sd 0.4] years; mean BMI SDS 1.9 [sd 0.4]. There were no significant differences in the number of minutes of outside play or television viewing a day between children in the intervention and the control condition. Also, the odds ratio for having breakfast daily or drinking two or less glasses of sweet beverages a day showed no significant differences between the two conditions. Additional analyses showed that the odds ratio for drinking less than two glasses of sweet beverages at follow-up compared with baseline was significantly higher for children in both the intervention (p < 0.001) and the control condition (p = 0.029).

**Conclusions:**

Comparison of the children in the two conditions showed that the intervention does not contribute to a change in health behaviors. Further studies are needed to investigate opportunities to adjust the intervention protocol, such as integration of elements in the regular well-child visit. The intervention protocol for youth health care may become part of a broader approach to tackle childhood overweight and obesity.

**Trial registration:**

Current Controlled Trials ISRCTN04965410

## Background

The prevalence of childhood overweight and obesity has been increasing over recent years [1]. The prevalence of overweight among children in the Netherlands has been estimated at 13-15% and the prevalence of obesity was estimated at 2% (age 2–21 years) [2]. The consequences associated with overweight and especially obesity in childhood (e.g. type 2 diabetes, cardiovascular disease) represent a public health issue [3,4]. Worldwide, interventions aiming to prevent overweight and obesity among children have been developed and evaluated [5].

In the Netherlands, growth, development and health of all children (0–19 years) is monitored in a nationwide program with regular well-child visits at set ages by providers of preventive youth health care. In each Dutch region youth health care providers (mainly youth health care physicians and school nurses) work in teams at youth health care centers and schools to conduct this nationwide program [6,7]. The program is offered free of charge by the government and participation is voluntary (attendance rate 90-100%) [8]. Several successful preventive measures have been implemented through the youth health care, for example, the national immunization program and the prevention of Sudden Infant Death Syndrome (SIDS) [9-11].

In 2004, a practiced-based protocol was developed to help detect overweight and obesity among children attending a well-child visit [12,13]. By means of this detection-protocol children were classified into weight categories using the international age-and-sex specific body mass index (BMI) cut-off values [14]. In 2005, a transitional plan, i.e. the prevention protocol, was developed to be used in daily practice [15]. This prevention protocol describes a set of actions that can be undertaken by the youth health care professional after the weight category of the child has been determined. Parents of and children with obesity are to be referred to the general practitioner. Parents of children with overweight can be offered additional healthy lifestyle counseling to prevent the children from developing obesity. With this intervention parents are supported in becoming aware of the overweight of their child, and motivated and assisted in making behavioral change. Parents play an important role in the child’s health behavior by performing certain parenting practices (e.g. rules with regard to eating snacks) and influencing the home environment (e.g. availability of (un)healthy products) [16,17]. During the healthy lifestyle counseling, advice is given about behaviors relevant to the prevention of overweight, e.g. having breakfast, drinking less sweet beverages, playing outside, and watching less television [15]. The detection and prevention protocol has the potential to reach a considerable number of parents and children and to be structurally implemented in the youth health care setting. Consequently, even if individual changes are small, greater effects on the population level might be achieved.

In this study we evaluate the effects of the intervention described in the prevention protocol focusing on preventing overweight children from developing obesity. We earlier reported that no effects of this intervention protocol were found on BMI and waist circumference of children in the total study population [18]. Therefore, this study evaluates changes in health behaviors targeted with the intervention protocol [19]. We hypothesized that children with overweight (not obesity) whose parents received advice according to the intervention protocol would, at follow-up, have breakfast more often, drink less sweet beverages per day, play outside more often, and watch less television per day compared to overweight children whose parents received usual care during the regular well-child visit. In addition, we evaluated the effects of the intervention protocol on related health behaviors (e.g. snacking, fruit consumption), parenting practices and home environment characteristics. Finally, implementation of the intervention protocol (e.g. health behaviors discussed) was also evaluated.

## Methods

The 'Be active, eat right’ study (trial registration Current Controlled Trials ISRCTN04965410) is a cluster randomized controlled trial (RCT) described in detail elsewhere [19]. The Medical Ethics Committee of the Erasmus University Medical Center Rotterdam approved the study protocol (reference number MEC-2007-163).

In 2007 all youth health care centers in the Netherlands (n = 37) were invited to participate in the study. The main eligibility criteria was that each center participated with both an intervention and a control condition with one or more youth health care teams. Also, eligible youth health care centers had not yet implemented the prevention protocol as usual care. Nine centers were eligible and agreed to participate, with a total of 44 youth health care teams [19]. Within each youth health care center, youth health care teams (with youth health care physician, school nurse and assistant) were randomized for allocation to either the intervention or control condition.

All parents invited to attend the well-child visit of their 5-year-old child between September 2007 and October 2008 were also invited to participate in the study with their child. Information on the study and an informed consent form for participation in the two-year study was enclosed with the invitation for the well-child visit. The parents could return the written informed consent form during the well-child visit when their child was 5 years old. Parents were not aware of the research condition to which they were allocated.

### Intervention

The intervention protocol is based on theories and models of behavioral change, i.e. the ASE model, a theoretical model of exercise habit formation, the Precaution Adoption Process Model, the Elaboration Likelihood Model, the stages of change model, and motivational interviewing techniques [20-26]. During the well-child visit and in up to three additional visits, parents of overweight children were offered tailored information regarding a healthy lifestyle [14,15].

Youth health care professionals were provided with a half-day training in motivational interviewing techniques [15,21,26]. Youth health care professionals could make use of these motivational interviewing techniques to create awareness, motivate parents to change behavior and/or support behavioral change. Before the training the professionals received a workbook with information on theories of behavior change and practical examples of interviews with parents. During the training the workbook was discussed and professionals were actively involved in exercises with an actor to practice motivational interviewing techniques.

Together with the research materials a hand-out was distributed among the youth health care professionals at the start of the study summarizing how the information from the training could step-by-step be applied during the well-child visit and additional intervention sessions. Also, materials were provided to support the professionals, for example, youth health care professionals could use a quick scan to map child health behavior or provide parents with a diary to increase parental insight in their child’s health behavior.

Based on the international literature and expert meetings, four lifestyle-related behaviors are described in the prevention protocol to promote the development of a healthy weight: i) playing outside for at least 1 h a day, ii) having breakfast daily, iii) drinking no more than 2 glasses of sweet beverages, and iv) limiting television time to a maximum of 2 h a day [15,27-31]. These behaviors are the focus of the intervention protocol for overweight children and their parents. Together with the parents, youth health care professionals chose one or two behaviors to target during the well-child visit and/or the additional intervention sessions. Parents drew up a family-oriented action plan on the health behavior change they wanted to achieve in their family.

When a child with overweight (not obesity) visited a youth health care team allocated to the control condition, usual care was given: i.e. the well-child visit, during which parents were offered general information about healthy nutrition and physical activity.

### Data collection

Data collection was scheduled at enrolment, baseline (the well-child visit), and at 2 years post-baseline (follow-up). A baseline questionnaire was enclosed with the regular invitation for the well-child visit. Parents could return the baseline questionnaire during the well-child visit when their child was 5 years old. Two years after the well-child visit, parents received an invitation to fill in a questionnaire, which could be completed by paper or via the Internet.

### Outcomes

The primary outcomes of the study, as described by Veldhuis et al. [19], were BMI and waist circumference, as reported elsewhere [18]. Here we report on the secondary outcome measures: child health behaviors. Questionnaires used in related research were used to assess outcomes [32-35]. Power calculations used to calculate the power to detect a difference between both conditions are reported by Veldhuis et al. [19]. Details on the variables and their psychometric properties are available in the Additional file 1: Table S1.

*Child health behavior (breakfast, sweet beverages, playing outside and TV viewing)* was assessed by parent report by means of questionnaires; parents had to keep in mind an average week when reporting on the health behaviors of their child.

Parents reported the number of days the child had breakfast (never to 7 days a week). Parents reported the average number of glasses of sweet beverages per day; examples of sweet beverages were given (e.g. soda, lemonade, fruit juice). Due to the categorical response scales and the distribution of data, these variables were dichotomized: drinking >2 glasses of sweet beverages a day vs. drinking ≤2 glasses a day, and having breakfast daily vs. not having breakfast daily.

The average number of days of screen time of the child during the week and the weekend was reported by parents; they also estimated the time spent in front of the screen (including DVD viewing) in hours and minutes on both a week day and weekend day. An estimated screen time in minutes per day was calculated. Time playing outside was assessed in a similar manner. Outside play and TV viewing were also dichotomized: playing outside <1 h a day vs. playing outside ≥1 h per day, and watching television >2 h a day vs. watching television ≤2 h a day.

### Outcomes measured at follow-up

#### Related child health behaviors

How many days the child walked and bicycled to school was reported by parents and dichotomized into 'never’ vs. 'once or more a week’. The time the child spent exercising at sports clubs and the time spent behind the computer or game console was reported; minutes of sports per week and minutes of computer time per day were calculated.

Parents estimated daily child consumption of snacks, in between meals, (1 serve = 1 piece), fruit (1 serve = one medium apple, banana or pear) and vegetables (1 serve = 1 serving spoon). Also, the child’s consumption of water or tea without sugar was estimated by parents and thereafter dichotomized into < 2 glasses a day vs. ≥ 2 glasses a day.

#### Parenting practices

Parents indicated whether they had rules for the child (yes vs. no) for 12 health behaviors: 8 behaviors were classified as healthy behaviors and 4 as unhealthy behaviors. A typical question is "Do you have rules at home about what your child can eat for breakfast?". Two indexes for rules were created, one for healthy and one for unhealthy behavior, by adding the number of times a parent answered 'yes’.

Whether the parent monitored child behavior was assessed with 10 items: 6 on healthy and 4 on unhealthy behaviors. A typical question was "How often do you monitor how much vegetables your child eats?". Questions were accompanied by a 5-point response scale ranging from 'never’ to 'always’. The items were combined by adding up scores and dividing by the number of items in the scale ro calculate an average score for healthy (Cronbach’s α 0.71) and unhealthy behaviors (Cronbach’s α 0.77) separately (scale range 1–5).

Similarly, also assessed was whether the parent actively encouraged healthy behavior (6 items), or discouraged unhealthy behavior (4 items), by saying this to the child (e.g. go play outside, or do not drink sweet beverages). An encouraging (Cronbach’s α 0.90) and discouraging (Cronbach’s α 0.79) scale score was calculated (scale range 1–5).

The number of days the parent had breakfast together with the child (1–7 days), how often parents collected food from a fast-food restaurant (ranging from 'never’ to 'every day’ on a scale from 0–7) and parental television time (minutes per day) was reported.

#### Home environment characteristics

Home environment included the availability of healthy and unhealthy products, reported by parents on a scale from 1–5, with a higher score indicating more healthy (Cronbach’s α 0.78) or more unhealthy (Cronbach’s α 0.67) products available.

#### Intervention implementation evaluation

The youth health care professionals from the intervention and control condition teams were to return a registration form after the well-child visit. The youth health care professionals in the intervention condition also returned a registration form after each additional intervention session. These forms addressed the duration of each intervention session, the topics discussed during the intervention session (e.g. overweight in general, playing outside, having breakfast, sweet beverages, watching television), whether action plans for change or workbook exercises regarding health behaviors were discussed, and whether a new additional intervention session was planned.

### Other measures

#### Child measures

Body weight, height and waist circumference were measured by the healthcare professionals during well-child visits using standardized methods as described in a protocol [12]. Child BMI was calculated by dividing weight in kilograms by squared height in meters. Child BMI Standard Deviation Scores (SDS) were calculated using the reference population of children from the 2009 Dutch National Growth study [2]. Children were classified as having normal weight, overweight (not obesity) or obesity according to the international age- and sex specific cut-off points for BMI [36].

Information on the child’s age (months) was obtained from the well-child visit registration. Information on child’s sex (male, female) and ethnic background (Dutch, non-Dutch) was obtained at enrollment by parent report. The child’s ethnic background was categorized according to the parents’ country of birth: if both parents were born in the Netherlands the child was classified as 'Dutch’ otherwise the child was classified as 'non-Dutch’ [37].

#### Maternal measures

The majority of the questionnaires was completed by mothers (88.1%). Information on maternal age (years), height (meters), weight (kilograms), country of birth (the Netherlands, other countries) and educational level (low, mid-low, mid-high, high) was self-reported in the baseline questionnaire. Maternal BMI was calculated and dichotomized into normal weight (BMI <25 kg/m^2^) or overweight (BMI ≥25 kg/m^2^) [38]. Maternal education level consisted of four categories: low (no education, primary school, or ≤3 years of general secondary school) mid-low (>3 years of general secondary school), mid-high (higher vocational training, undergraduate programs, Bachelor s degree) or high (higher academic education) [39].

### Statistical analysis

Baseline socio-demographic characteristics of the intervention and control condition clusters are described using descriptive statistics. Additional file 2: Table S2 presents the baseline characteristics of all participants (n = 8,784).

For each research condition, we evaluated whether health behavior made a significant change between baseline and follow-up. A cluster-corrected, intercept only, linear regression model was fitted for television viewing and outside play (minutes per day). For having breakfast daily and drinking ≤ 2 glasses of sweet beverages a day, cluster-corrected McNemar tests were performed (R software, R 2.7.1, Development Core Team, Vienna, Austria [40]).

To predict follow-up health behaviors and compare research conditions, regression models were fitted. All participants were analyzed according to the intention-to-treat principle. All models are presented with and without correction for clustering at youth health care team level (n = 44) [41]. A two-predictor model was fitted: research condition (intervention or control) and baseline value of the outcome variable [42]. Time between baseline and follow-up measurement was added to the model (mean 26.0 [sd 4.42] months, range 14–35 months) and age at baseline measurement was added to the model (mean 69.6 [sd 5.18] months, range 56.4-91.2). Chi-square tests showed that the distribution of the months in which the questionnaires at baseline and follow-up were completed was comparable between both research conditions. Analyses corrected for season were performed (data not shown) and results were comparable to the analyses without correction. In all analyses, the effect of the intervention was evaluated at the p < 0.05 level. Interaction effects between research condition and socio-demographic characteristics (sex and ethnic background of the child, education level of the mother) were explored [19]. The interaction terms were considered statistically significant at p < 0.10 [43].

Additional analyses included an evaluation of related health behaviors, parenting practices and the home environment characteristics measured at follow-up, by means of cluster-corrected regression analysis. The analyses were similar to the main health behavior outcomes, except that there was no correction for the baseline value of the outcome. A cluster-corrected per protocol analysis was performed predicting health behaviors at follow-up with regression analysis comparing overweight children of parents that attended one or more additional intervention session (n = 138), two or more additional intervention sessions (n = 97) and three additional intervention sessions (n = 55) with overweight children in the control condition (n = 288) (Additional file 2: Table S2). Also, we performed explorative analyses to evaluate the effects of the intervention based on the behavior that was discussed during the well-child visit. For example, the effects on breakfast for the subgroup of children in the intervention condition in which the parents discussed breakfast; these children were compared with children in the control condition. Analyses were corrected for cluster (Additional file 3: Table S3).

Demographic characteristics (age, country of birth, education level, overweight) of mothers attending at least one additional intervention session were compared with characteristics of mothers receiving no additional intervention sessions by means of descriptive statistics. Also, descriptive statistics were used to describe implementation of the intervention protocol from the registration data of the youth health care professionals.

Analyses without cluster correction were performed in SPSS (International Business Machines (IBM) Corp., SPSS statistics, version 20.0, Armonk, NY, USA). Linear and logistic regression analyses, taking into account the clustered design of the study, were performed using SAS software (SAS version 9.2; SAS Institute Inc, Cary, NC, USA).

## Results

**Figure 1:**
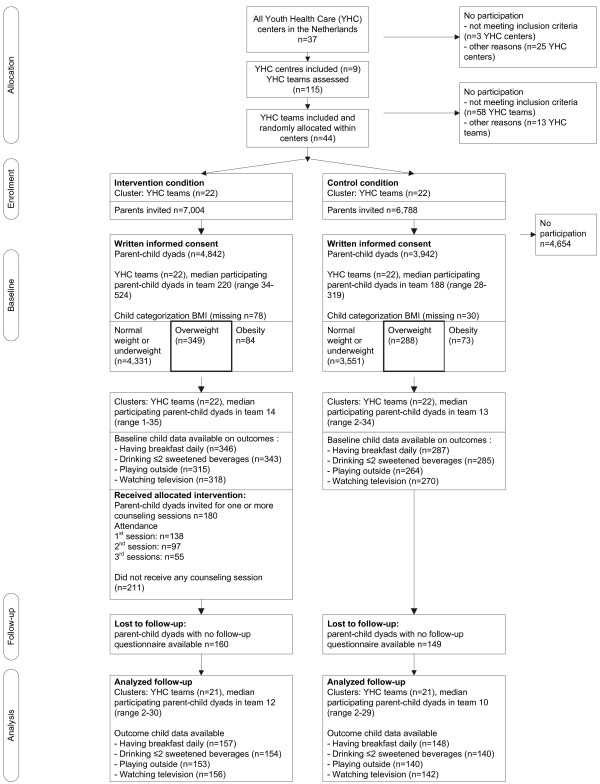
Flow diagram of the selection and follow-up of study participants.

Figure 1 presents the flow of clusters and participants through the study. The intervention protocol was to be offered to parents with children with overweight, not obesity, at baseline (n = 637). Due to missing data at follow-up (i.e. no return of the questionnaire), the population for analysis for having breakfast was n = 305, for sweet beverages n = 294, for television viewing n = 298 and for playing outside n = 293 (Figure 1).

### Characteristics of children with overweight, not obesity (n = 637) at baseline

At baseline, 38.1% of the children was male and the mean age was 69.09 (sd 5.18) months (Table 1). Mothers had an average age of 35.9 (sd 4.3) years and 16.9% was born outside the Netherlands.

**Table 1:** Descriptive socio-demographic characteristics of the study sample at baseline (n = 637)

	**Total sample (n = 637)**	**Intervention condition (n = 349)**	**Control condition (n = 288)**	p-value*
**Child characteristics**				
Age, months (SD) [n = 0 missing]	69.09 (5.18)	68.65 (4.98)	69.64 (5.37)	**0.016**
Sex, % boys [n = 0 missing]	38.1	38.7	37.5	0.412
Ethnic background, % Dutch [n = 11 missing]	78.0	75.8	80.6	0.091
BMI SDS (SD)§ [n = 0 missing]	1.90 (0.37)	1.93 (0.38)	1.88 (0.35)	0.087
**Parental characteristics**				
**Mothers**				
Age, years (SD) [n = 81 missing]	35.85 (4.29)	35.80 (4.23)	35.92 (4.37)	0.741
Country of birth, % the Netherlands [n = 4 missing]	83.1	82.4	84.0	0.335
Education level, % [n = 6 missing]				
Low	7.3	7.8	6.6	0.238
Low to mid-low	26.0	27.0	24.8	0.086
Mid-high to high	44.7	40.0	50.3	0.721
High	22.0	25.2	18.2	**0.003**
BMI, % overweight [n = 51 missing]	44.0	44.5	43.4	0.422
**Fathers**				
Age, years (SD) [n = 562 missing]	40.19 (7.35)	40.60 (7.87)	39.71 (6.78)	0.606
Country of birth, % the Netherlands [n = 9 missing]	83.6	80.8	87.0	**0.022**
Education level, % [n = 18 missing]				
Low	5.3	4.5	6.4	0.602
Low to mid-low	26.0	25.3	26.9	0.478
Mid-high to high	42.5	42.6	42.4	0.156
High	26.2	27.7	24.4	0.059
BMI, % overweight [n = 252 missing]	66.5	66.3	66.7	0.514

*p-value from *t*-test for continuous variables evaluating the differences in means between intervention and control condition. P-value from Chi-square test for categorical values, evaluating the differences between frequencies between intervention and control condition.

§BMI SDS: reference data from the 2009 Dutch National Growth Study.

Note: **Bold** printed p-values indicate statistically significant difference between intervention and control condition.

### Health behavior outcomes

Table 2 presents descriptive statistics of the health behaviors and results of the logistic regression analyses. At follow-up, in both conditions a significantly higher percentage of children played outside for < 1 h and watched >2 h of TV compared with baseline. At follow-up, in the control condition the percentage of children eating breakfast daily was higher than at baseline (p = 0.027). In both conditions, at follow-up a higher percentage of children was drinking ≤ 2 sweet beverages a day compared with baseline (Table 2).

**Table 2:** Baseline and follow-up percentages of health behaviors and regression coefficients of the intervention condition compared with the control condition

	**n**	**Intervention condition**	**n**	**Control condition**	**Odds ratio (95% CI)**^**1**^	**Odds ratio (95% CI)**^**2**^
**Having daily breakfast**						
Baseline	346	89.9%	287	88.2%*		
Follow-up	157	95.5%	148	94.6%*	1.04 (0.29; 3.75)	1.04 (0.28; 3.78)
**Drinking ≤ 2 sweet beverages a day**						
Baseline	343	32.1%***	285	33.3%*		
Follow-up	154	55.2%***	140	47.9%*	1.38 (0.84; 2.26)	1.38 (0.84; 2.27)
**Outside play ≥ 1 hour a day**						
Baseline	315	93.3%***	264	94.3%**		
Follow-up	153	77.1%***	140	77.1%**	1.11 (0.60; 2.06)	1.09 (0.53; 2.26)
**Watching television ≤ 2 hours a day**						
Baseline	318	74.8%*	270	75.2%*		
Follow-up	156	66.0%*	142	69.0%*	0.93 (0.54; 1.61)	0.93 (0.53; 1.61)

^1^Odds ratio (OR) and 95% confidence interval (CI) from regression model evaluating the difference between intervention and control condition on the outcome at follow-up, corrected for baseline distribution of the outcome, time between measurements and age of the child at baseline.

^2^Odds ratio (OR) and 95% confidence interval (CI) from multilevel regression model evaluating the difference between intervention and control condition on the outcome at follow-up, corrected for baseline distribution of the outcome, time between measurements and age of the child at baseline.

Note: **Bold** printed numbers indicate statistically significant within-group change of behavior; within condition change of behavior from baseline to follow-up was evaluated using cluster-adjusted McNemar analysis, asterisks indicates significance level: *p < 0.05, **p < 0.01, ***p < 0.001.

At follow-up there were no significant differences between the two conditions with regard to having breakfast, drinking sweet beverages, playing outside or viewing television. Also, there was no significant difference at follow-up between the two conditions when comparing outside play and television viewing using linear regression analysis (change in minutes per day) (Table 3).

**Table 3:** Baseline and follow-up means of playing outside and watching television per day, and regression coefficients of the intervention condition compared with the control condition

	**n**	**Intervention condition**	**n**	**Control condition**	**Beta coefficient (95% CI)**^**1**^	**Beta coefficient (95% CI)**^**2**^
**Outside play, mean (SD)**						
Baseline	315	161.84 (106.66)*	264	160.50 (100.91)		
Follow-up	153	135.93 (91.32)*	140	123.82 (76.34)	15.00 (-4.14; 34.15)	8.22 (-15.77; 32.22)
**Watching television, mean (SD)**						
Baseline	318	103.93 (74.05)	270	105.94 (66.57)		
Follow-up	156	102.64 (61.67)	142	104.34 (54.81)	-1.56 (-14.56; 11.44)	-1.56 (-14.57; 11.45)

^1^Beta coefficient and 95% confidence interval (95% CI) from regression model evaluating the difference between intervention and control condition on the outcome at follow-up, corrected for baseline value of the outcome, time between measurements and age of the child at baseline.

^2^Beta coefficient and 95% confidence interval (95% CI) from multilevel regression model evaluating the difference between intervention and control condition on the outcome at follow-up, corrected for baseline value of the outcome, time between measurements and age of the child at baseline.

Note: **Bold** printed numbers indicate statistically significant within-group change; within-group change of behavior from baseline to follow-up was evaluated using cluster-adjusted intercept only regression analysis, asterisks indicates significance level: *p < 0.05.

The per protocol analyses, comparing children of parents that received at least one, two or three additional intervention sessions with the control condition, showed that children of parents receiving 3 or more additional intervention sessions, had a significantly higher OR for drinking ≤ 2 sweet beverages a day (p < 0.05) (Additional file 3: Table S3).

The explorative analyses indicated that children of parents discussing sweet beverages during the well-child visit had a higher OR for drinking ≤ 2 sweet beverages at follow-up compared with children in the control condition, almost reaching significance (p < 0.10) (Additional file 4: Table S4). With regard to the other health behaviors, parents in the intervention condition had a significantly lower OR of having children watching ≤ 2 hours of television (p < 0.05) when this behavior was discussed.

### Evaluation of potential interaction

Three significant interactions were observed: sex and intervention condition when predicting outside play in minutes per day (p = 0.019) and sweet beverage consumption (p = 0.038), and education level and intervention condition in the model predicting minutes of television viewing a day (p = 0.011). Stratified analyses were performed. Boys in the intervention condition had an OR of 2.74 (95% CI 1.19-6.35) and girls had an OR of 0.93 (95% CI 0.40-1.77) to drink ≤ 2 sweet beverages a day. Boys in the intervention condition had a change in outside play of -13.99 min/day (95% CI -46.11 to 18.13) while girls in the intervention condition had a change in outside play of 31.65 min/day (95% CI 4.32-58.98). Education level was merged into two categories (low/mid-low and mid-high/high) to perform stratified analyses. No significant results were observed when evaluating the effects on TV viewing for children of mothers with low/mid-low or mid-high/high education level.

### Evaluation of related health behaviors, parenting practices and home environment characteristics

Table 4 presents descriptive characteristics and results of the regression analyses for the related health behaviors, parenting practices and home environment characteristics. There were no significant differences between children in the two conditions with regard to related health behaviors (Table 4). With regard to parenting practices, parents of children in the intervention condition appeared to have more rules with regard to lifestyle behaviors compared with the control condition (rules on healthy behaviors mean 6.1 (sd 2.0) vs. 5.8 (sd 1.9) (p = 0.030), and rules on unhealthy behaviors mean 3.2 (sd 1.2) vs. 2.3 (sd 1.3) (p = 0.009) (Table 4). There were no significant differences between the two conditions for the remaining parenting practices or home environment characteristics (Table 4).

**Table 4:** Descriptive statistics and regression analyses predicting secondary health behavior outcomes of the child, parenting practices and home environment characteristics at follow-up

	**n**	**Intervention condition**	**n**	**Control condition**	**Beta coefficient/Odds ratio (95% CI)**^**1**^
**Health behaviors**					
Walk to school (once or more a week)	159	52.8%	145	49.7%	1.12 (0.63; 1.98)
Bicycle to school (once or more a week)	158	65.8%	148	64.2%	1.15 (0.60; 2.21)
Average time spent performing sports (min/week, mean [sd])	128	113.83 [60.67]	115	115.43 [64.21]	1.68 (-14.34; 17.69)
Computer games (min/day, mean [sd])	144	29.61 [28.22]	144	30.62 [36.76]	-2.98 (-11.99; 6.03)
Candy and snacks (pieces/day, mean [sd])	151	1.06 [0.45]	140	1.09 [0.65]	-0.03 (-0.16; 0.11)
Vegetables (spoons/day, mean [sd])	157	1.61 [0.87]	144	1.72 [0.99]	-0.13 (-0.36; 0.10)
Fruit (pieces/day, mean [sd])	154	1.39 [0.88]	138	1.41 [0.91]	-0.01 (-0.29; 0.27)
Water or tea without sugar (≥2 per day)	144	25.7%	127	22.0%	1.24 (0.63; 2.43)
**Parenting practices**					
Family breakfast (days/week, mean [sd])	158	5.46 [2.03]	145	5.25 [2.11]	0.52 (-0.15; 1.20)
Eating outside the home (days/week, mean [sd])	156	0.26 [0.18]	142	0.29 [0.24]	-0.04 (-0.09; 0.01)
Rules (number of rules, mean[sd])					
Healthy behavior (range 0–8)	159	6.24 [1.84]	147	5.83 [1.89]	0.52 (0.05; 0.99)*
Unhealthy behavior (range 0–4)	158	3.35 [0.99]	143	2.96 [1.19]	0.43 (0.11; 0.75)**
**Parenting practices**					
Monitoring (range 1–5, mean [sd])					
Healthy behavior	157	4.56 [0.41]	142	4.53 [0.43]	0.04 (-0.07; 0.14)
Unhealthy behavior	156	4.23 [0.63]	145	4.19 [0.63]	0.06 (-0.11; 0.24)
Reinforcing/discouraging (range 1–5, mean [sd])					
Healthy behavior	153	2.89 [1.22]	135	2.89 [1.17]	-0.07 (-0.42; 0.28)
Unhealthy behavior	151	3.15 [0.87]	140	2.98 [0.79]	0.12 (-0.13; 0.37)
Parental TV viewing (min/day, mean [sd])	155	133.75 [87.27]	146	125.53 [76.50]	11.93 (-9.30; 33.17)
**Home environment**					
Healthy products available (range 1–5, mean [sd])	159	4.79 [0.35]	145	4.82 [0.33]	-0.02 (-0.11; 0.06)
Unhealthy products available (range 1–5, mean [sd])	159	4.05 [0.80]	148	4.17 [0.81]	-0.09 (-0.34; 0.16)

^1^Beta coefficient or odds ratio (95% confidence interval) for the intervention condition versus control condition (reference) at follow-up from regression model adjusted for cluster, corrected for time between measurements and age at baseline.

Note: **Bold** printed numbers indicate statistically significant difference between groups; asterisks indicates significance level: *p < 0.05, **p < 0.01.

### Evaluation of intervention implementation

The youth health care professionals performing the well-child visit and the additional intervention sessions in the intervention condition were mainly youth health care physicians (72.0% and 65.8%). Attendance at the first additional intervention session was 76.7% (138/180), the second 53.9% (97/180) and at the third 30.6% (55/180) (Figure 1). Average duration of the first additional intervention session was 24.8 [sd 10.5] minutes. The baseline BMI SDS of children whose parents attended at least one additional intervention session was higher than that of children whose parents did not attend any additional intervention session (mean BMI SDS 1.99 [sd 0.35] vs. 1.89 [sd 0.0.394], p = 0.011). Mothers that attended at least one additional intervention session (n = 138) showed no significant difference with regard to age, country of birth, education level or BMI compared with mothers that did not attend any additional intervention session.

During the well-child visit youth health care professionals most often advised parents with regard to drinking less sweet beverages (134/349, 38.4%) and playing outside more often (104/349, 29.8%). During the first additional intervention session, most attention was given by the youth health care professionals to creating awareness and knowledge with regard to overweight (86/138, 62.3%) and the four health behaviors targeted in with the intervention protocol (78/138, 56.5%). Physicians also reported that they motivated parents to change health behaviors during the first additional intervention session (73/138, 52.9%). During the first additional intervention session youth health care professionals most often made an appointment with the parents about changing the amount and type of sweet beverages consumed by the child (78/138, 56.5%). In 41.3% (57/138) of the sessions diaries or work-book exercises were provided to the parents to be completed at home.

## Discussion

In this study we evaluated the effects of an intervention protocol implemented in youth health care. The goal of this intervention protocol was to change health behaviors related to overweight in 5-year-old children by providing their parents with low-intensive lifestyle counseling sessions. The results show no overall difference between children aged 5–7 years in the intervention and control condition with regard to changes in having breakfast, drinking sweet beverages, TV viewing and playing outside. This in in line with the lack of effects of the prevention protocol observed on measures of body composition: BMI and waist circumference, in the total study population [18].

Results indicate that in both conditions the behavior changed in a similar direction from baseline to follow-up. The national release of the prevention protocol, of with the intervention protocol is an element, received enthusiasm of the youth health care professionals; they received tools to contribute to the promotion of a healthy weight and lifestyle among children. Specifically for the evaluation of the intervention protocol, this might have influenced the care professionals provided. Although the control condition teams were instructed to offer usual care, professionals allocated to the control condition may have provided "improved" usual care to the parents, consequently stimulating health behavior change. Also, the low attendance of parents to the optional additional intervention sessions may have diminished the contrast between both conditions.

However, in both conditions, a significant healthful change in sweet beverage consumption was observed between baseline and follow-up. Together with the youth health care professional, parents chose the health behavior they thought most feasible to change and, therefore, only one or two behaviors were discussed during the additional intervention sessions [15]. In line herewith, most often sweet beverage consumption of the child was discussed. Sweet beverage consumption is associated with overweight; interventions may effectively decrease sweet beverage consumption and thus body weight of children [44,45]. The intervention protocol may have increased awareness among parents about the amount of sugar in sweet beverages, or alternative non-sweet beverages to offer the child.

The evaluation of the intervention protocol was performed in youth health care and the current results may reflect the use of this protocol in its current design [15]. It may be worthwhile to investigate improvements of elements of the protocol to enhance implementation and use, because of the reach of youth health care among parents and children [46,47]. According to the youth health care professionals that provided the intervention protocol, it is difficult to motivate parents to change health behavior and attend an additional session [18]. The involvement of parents is essential for successful overweight prevention, and especially valuable in interventions targeting young children [48]. Lack of attendance is often observed in interventions performed in a primary care setting [49,50]. Motivational interviewing is reported to be effective in increasing motivation and participation of parents [51-54]. The youth health care professionals’ training in this study provided elements of motivational interviewing techniques; we recommend to assess whether more training of professionals is needed to further improve these skills and apply them more effectively in daily practice [51,55]. Observational research during the well-child visit and the additional intervention sessions may be recommended to evaluate the use of motivational interviewing techniques by the youth health care professionals. Qualitative research with parents is recommended to assess what motivates parents to change their child’s health behavior.

Another opportunity to improve the intervention protocol may be elaboration of its implementation during the well-child visit; this visit was well attended by parents. Adherence to additional intervention sessions may be improved by using e-mail, telephone messages and other types of modern communication techniques [56]. In addition, potentially, internet-based tailored elements may be used to improve adherence or to complement the information provided during the well-child visit or the additional intervention sessions [57]. Research is needed to evaluate whether these types of improvements to the intervention protocol have a positive impact on effects on health behaviors.

The present results are in contrast to the successful realization of an earlier immunization program and the large contribution to the prevention of SIDS by youth health care in the Netherlands [9-11]. However, both are different type of health issues with potentially more imminent and urgent effects. This is contrary to the complex problem of overweight [58]. Until now, few interventions performed in settings comparable to youth health care have been found effective [50,59-61]. In the Netherlands, youth health care performs regular height and weight checks and has the opportunity to offer parents and children individual, tailored advice and acts on the local level, which enables referral to local programs and care providers [7,8]. We recommend collaboration between youth health care and local intervention programs. This may provide opportunities to enhance effects and provide more children and parents with suitable intervention programs that help them to change health behavior [62]. Overall, tackling overweight as a public health problem should include more than this low-level intervention protocol but youth health care may contribute to the broader approach [13].

### Methodological considerations

Strengths of this study include the large number of youth health care centers willing to participate and apply the intervention protocol in daily practice. Moreover, we were able to evaluate the intervention protocol in the youth health care setting throughout the Netherlands, including a large number of parents and children. Consequently, we were able to detect the smaller number of overweight children and follow them for a period of two years.

Considerations with regard to implementation of the intervention protocol have been discussed elsewhere [18]. Other limitations include the missing data at follow-up; therefore, the present findings need to be interpreted with caution because a selective group of parents participated in the follow-up measurement. The items used in this study to assess health behavior and home-environmental characteristics need to be investigated with regard to their validity and reliability. Because parents reported on health behaviors of their child, we recommend complementing parent-report measures with objective and observational research to assess validation of parent report of child health behavior. Especially for physical activity behaviors, complementary measures (e.g. self-report questionnaires, accelerometers and GPS tracking) are recommended to investigate different types of physical activity behaviors.

Evaluation and reappraisal of the intervention protocol is needed to evaluate the opportunities of this program in youth health care to complement existing intervention initiatives. Further evaluation of the intervention protocol needs to include examination of potential side-effects on, e.g., health-related quality of life of the children.

## Conclusion

The prevention protocol describes a low-intensive intervention to change health behaviors associated with overweight and obesity for parents of overweight (not obese) children. This study evaluated the effects of the intervention protocol on 5-year-old children’s health behavior; similar changes were observed among children receiving usual care and children receiving the intervention protocol. However, a significant improvement (i.e. reduction) in sweet beverage consumption was observed in both conditions.

We recommend further research to evaluate the effect of adjustments and improvements of the intervention protocol (e.g. integrating elements in the regular well-child visit or higher parent participation in the additional sessions) on health outcomes. Qualitative research may be performed to gain insight into how to motivate parents to change health behavior. Collaboration between youth health care and intervention initiatives at local level is recommended to enhance effects and create a broad approach for the prevention and treatment of overweight and obesity.

## Abbreviations

SIDS: Sudden infant death syndrome; BMI: Body mass index.

## Competing interests

The authors declare that they have no competing interests.

## Authors’ contributions

HR and RH originated the study idea and were responsible for acquiring the study grant. CR conceptualized and designed the study and contributed to study coordination. AG and LV were responsible for data collection, study coordination, data analyses and reporting study results. AG was responsible for performing statistical analyses, drafting and revising the manuscript. CL designed the statistical analyses and contributed to performing statistical analyses and interpreting results. HR and RH were responsible for study supervision, overseeing data collection and reporting of study results. All authors contributed to interpretation of the data and critical revision of the manuscript for important intellectual content. All authors have read and approved the final manuscript.

## Pre-publication history

The pre-publication history for this paper can be accessed here:

http://www.biomedcentral.com/1471-2458/14/59/prepub

## Supplementary Material

Additional file 1: Table S1 Background information and psychometric properties of the variables. Click here for file

Additional file 2: Table S2 Descriptive characteristics of the children participating in the 'Be active, eat right’ study (n = 8,784). Click here for file

Additional file 3: Table S3 Evaluation of intervention effects based on 'dose’. Click here for file

Additional file 4: Table S4 Results from the regression analyses predicting health behavior outcomes based on the behavior discussed during the well-child visit. Click here for file
