# Supplementary material for: Promotion of a healthy lifestyle among 5-year-old overweight children: health behavior outcomes of the 'Be active, eat right’ study
Source: BMC Public Health. 2014 Jan 21;14:59. doi: 10.1186/1471-2458-14-59 (PMC3911965; doi:10.1186/1471-2458-14-59)
Supplement: Additional file 1: Table S1 — Background information and psychometric properties of the variables. [file 1471-2458-14-59-S1.docx]

**Table S1**. Background information and psychometric properties of the variables

| Variable item(s) | Response scale (assigned value) | Outcome in analyses | Label |
| --- | --- | --- | --- |
| **Child health behaviors** |  |  |  |
| How many days in the week does your child have breakfast? | 1(1), 2(2), 3(3), 4(4), 5(5), 6(6), 7(7) | <7 days a week vs. ≥7 days a week | Having daily breakfast |
| How many glasses of sweet beverages does your child consume on an average day? | Less than 1 (0.5), 1 to 2(1.5), 3 to 4(3.5), 5 to 6 (5.5), 7 to 8 (7.5), 9 or more (9.5) | ≤2 glasses vs. > 2 glasses | Drinking sweet beverages |
| On an average day how much time does your child play outside? | Open ended(hours/min) | Min/day and <1 hour a day vs. ≥1 hour a day | Outside play |
| On an average day how much time does your child watch TV? | Open ended(hours/min) | Min/day and ≤2 hours a day vs. > 2 hours a day | TV viewing |
|  |  |  |  |
| **Related child health behaviors** |  |  |  |
| How many days in the week does your child walk to school? | Never (0), 1 day(1), 2 days(2), 3 days(3), 4 days(4), 5 days(5) | Never vs. once or more a week | Waking to school |
| How many days in the week does your child bicycle to school? | Never(0), 1 day(1), 2 days(2), 3 days(3), 4 days(4), 5 days(5) | Never vs. once or more a week | Bicycle to school |
| How many hours a week does your child spent in organized sports activities? | Open ended(hours/min) | Min/week | Average time spent performing sports |
| How many days a week/ weekend does your child spent on the computer or game console?  How much time does your child spent on the computer or game console on an average week/weekend day? | Never(0), 1 day(1), 2 days(2)  Never (0), 1 day(1), 2 days(2), 3 days(3), 4 days(4), 5 days(5)  Open ended(hours/min)  Open ended(hours/min) | Min/week | Computer games |
| How many snacks / candies does your child eat on an average day? | None or less than 1(0.5), 1(1), 2(2), 3(3), 4(4), 5(5), 6(6), 7(7), 8(8), 9 or more(9.5) | Snacks and candy/day | Candy and snacks |
| How many serving spoons of vegetables does your child eat on an average day? | None (0), less than ½ a spoon (0.25), ½ a spoon(0.5), 1 spoon(1), 2 spoons(2), 3 spoons(3), 4 spoons(4), 5 spoons or more (5.5) | Spoons/day | Vegetables |
| How many pieces of fruit does your child eat on an average day? | None(0), less than ½ a piece(0.25), ½ an piece(0.5), 1 piece(1), 2 pieces(2), 3 pieces(3), 4 pieces(4), 5 pieces or more(5) | Pieces/day | Fruit |
| How many glasses of water or tea without sugar does your child consume on an average day? | None or less than 1(0.5), 1(1), 2(2), 3(3), 4(4), 5(5), 6(6), 7(7), 8(8), 9 or more(9.5) | ≤2 glasses vs. > 2 glasses | Water or tea without sugar |
|  |  |  |  |
| **Parenting practices** |  |  |  |
| Do you have rules in your home about:   - Whether your child has to play outside - Whether your child has to take part in physical activity - Whether your child should eat breakfast - What your child can and can’t eat with his/her breakfast - Having breakfast as a family at the table - How much and what kind of dinner your child can have - How often and how many snacks and candy your child can eat - How often and how many vegetables your child should eat - How often and many fruit your child should eat - How often and how many sweet beverages your child can drink - How often and how long your child can watch TV - How often and how long your child can sit behind the computer of game console | Yes (1), no(0) | Total rules healthy behaviors (n=8 items, score range 0-8)  Total rules unhealthy behaviors (n=4 items, score range 0-4) | Rules healthy behavior  Rules unhealthy behavior |
| Do you monitor whether your child:   - plays outside - is physically active - has breakfast daily - has dinner daily - eats snacks and candy - eats vegetables - eats fruit - drinks sweet beverages - watches TV - sits behind the computer/ game console | Never(1), rarely(2), sometimes(3), often(4), always(5) | Monitoring healthy behavior (n=6 behaviors, Cronbach’s α 0.71)  Monitoring unhealthy behavior (n=4 items, Cronbach’s α 0.77) | Monitoring healthy behavior  Monitoring unhealthy behavior |
| How often do you actively reinforce your child to:   - go play outside - go be physically active - have breakfast daily - have dinner daily - not eat snacks and candy - eat vegetables - eat fruit - not drinks sweet beverages - turn of the TV - turn of the computer/ game console | Never(1), rarely(2), sometimes(3), often(4), always(5) | Encouraging healthy behavior (n=6 items, Cronbach’s α 0.90)  Discouraging unhealthy behavior (n=4 items, Cronbach’s α 0.79)  Sum/ number of items | Reinforcing healthy behavior  Discouraging unhealthy behavior |
| How many days a week do you have breakfast with your child at the table? | 1(1), 2(2), 3(3), 4(4), 5(5), 6(6), 7(7) | Days/ week | Family breakfast |
| How many days a week does your family eat a mail from a fast food restaurant?  How many days a week does your family eat meals from McDonalds, KFC, Burgerking e.o?  How many days a week does your family eat pre-prepared meals (for example Chinese food or pizza delivery)? | Never(0), 1-3 times a months(0.5), 1 day a week(1), 2 days a week(2), 3 days a week(3), 4 days a week(4), 5 days a week(5), 6 days a week(6), every day(7). | Days/ week | Eating outside the home |
| How many days a week/ weekend do you watch television?  How much time do you watch television an average week/weekend day? | Never(0), 1 day(1), 2 days(2)  Never (0), 1 day(1), 2 days(2), 3 days(3), 4 days(4), 5 days(5)  Open ended (hours/min)  Open ended (hours/min) | Min/day | Parental TV viewing |
|  |  |  |  |
| **Home environment** |  |  |  |
| There are usually enough breakfast products available in our home to have breakfast  There are usually enough products in home to have dinner  There are usually snacks and candy products available in our home  There are usually vegetables available in our home  There is usually fruit available in our home  There are usually sweet beverages available in our home | Totally disagree(1), disagree(2), slightly agree/ slightly disagree(3), agree(4), totally agree (5) | Healthy products available (n=4 items, Cronbach’s α 0.78)  Unhealthy products available (n=2 items, Cronbach’s α 0.67)  Sum/ number of items | Healthy products available  Unhealthy products available |
|  |  |  |  |

Note: items used in this study are based on research performed by (Jaddoe, et al., 2006; Perez-Rodrigo, et al., 2005; Robinson, 1999; van der Horst, Oenema, van de Looij-Jansen, & Brug, 2008; Wendel-Vos, Schuit, Saris, & Kromhout, 2003)

**References**

Jaddoe, V. W., Mackenbach, J. P., Moll, H. A., Steegers, E. A., Tiemeier, H., Verhulst, F. C., et al. (2006). **The Generation R Study: Design and cohort profile**. *Eur J Epidemiol, 21*(6), 475-484.

Perez-Rodrigo, C., Wind, M., Hildonen, C., Bjelland, M., Aranceta, J., Klepp, K. I., et al. (2005). **The pro children intervention: applying the intervention mapping protocol to develop a school-based fruit and vegetable promotion programme**. *Ann Nutr Metab, 49*(4), 267-277.

Robinson, T. N. (1999). **Reducing children's television viewing to prevent obesity: a randomized controlled trial**. *Jama, 282*(16), 1561-1567.

van der Horst, K., Oenema, A., van de Looij-Jansen, P., & Brug, J. (2008). **The ENDORSE study: research into environmental determinants of obesity related behaviors in Rotterdam schoolchildren**. *BMC Public Health, 8*, 142.

Wendel-Vos, G. C., Schuit, A. J., Saris, W. H., & Kromhout, D. (2003). **Reproducibility and relative validity of the short questionnaire to assess health-enhancing physical activity**. *J Clin Epidemiol, 56*(12), 1163-1169.
