# Supplementary material for: Promotion of a healthy lifestyle among 5-year-old overweight children: health behavior outcomes of the 'Be active, eat right’ study
Source: BMC Public Health. 2014 Jan 21;14:59. doi: 10.1186/1471-2458-14-59 (PMC3911965; doi:10.1186/1471-2458-14-59)
Supplement: Additional file 2: Table S2 — Descriptive characteristics of the children participating in the 'Be active, eat right’ study (n = 8,784). [file 1471-2458-14-59-S2.doc]

**Table S2** Descriptive characteristics of the children participating in the ‘Be active, eat right’ study (n=8,784)

|  | Overall  (n=8,784) | Intervention condition  (n=4,842) | Control condition  (n=3,942) | *p-value** |
| --- | --- | --- | --- | --- |
| **Child characteristics** |  |  |  |  |
| Mean age (sd), months  (missing n=38) | 68.93 (5.07) | 68.86 (5.09) | 69.03 (5.06) | 0.121 |
| Sex (% boys)  (missing n=76) | 50.9 | 51.7 | 50.0 | 0.055 |
| Ethnical background (% Dutch)  (missing n=176) | 84.9 | 83.9 | 86.2 | **0.002** |
| Mean BMI (sd), kg/m²  (missing n=34) | 15.49 (1.52) | 15.53 (1.49) | 15.43 (1.54) | **0.002** |
| Mean BMI SDS (sd)§  (missing n=72) | 0.10 (1.11) | 0.14 (0.02) | 0.05 (1.14) | **<0.001** |
|  |  |  |  |  |
| **Mothers’ characteristics** |  |  |  |  |
| Mean age (sd), years  (missing n=1,116) | 36.30 (4.46) | 36.31 (4.50) | 36.28 (4.41) | 0.786 |
| Country of birth (% the Netherlands)  (missing n=108) | 89.4 | 88.8 | 90.1 | **0.023** |
| Education level  (missing n=167) |  |  |  | **0.046** |
| Low / Mid-low | 23.6 | 24.3 | 22.8 |  |
| Mid-high/ High | 76.4 | 75.7 | 77.2 |  |
| BMI categories  (missing n=921) |  |  |  | **0.018** |
| Normal weight | 70.1 | 69.1 | 71.3 |  |
| Overweight/ obesity | 29.9 | 30.9 | 28.7 |  |

* p-value derived from Chi-square tests comparing intervention and control condition on categorical and binomial outcomes, p-value derived from independent samples t-test comparing intervention and control condition on continuous outcomes.

§ BMI SDS: reference data from the 2009 Dutch National Growth Study

Note: **bold** printed numbers indicate significant p-value
