# Supplementary material for: Promotion of a healthy lifestyle among 5-year-old overweight children: health behavior outcomes of the 'Be active, eat right’ study
Source: BMC Public Health. 2014 Jan 21;14:59. doi: 10.1186/1471-2458-14-59 (PMC3911965; doi:10.1186/1471-2458-14-59)
Supplement: Additional file 3: Table S3 — Evaluation of intervention effects based on 'dose’. [file 1471-2458-14-59-S3.docx]

**Table S3** Evaluation of intervention effects based on ‘dose’

|  | **Number of additional sessions** |  |  |
| --- | --- | --- | --- |
|  | **1 or more (n=138)** | **2 or more (n=97)** | **3 or more (n=55)** |
| **Continues** | *Beta (95%CI)* | *Beta (95%CI)* | *Beta (95%CI)* |
| Playing outside (min/day) | 17.53 (-8.76; 43.83) | 21.75 (-6.22; 49.72) | 32.90 (-0.33; 66.14) |
| TV viewing (min/day) | 5.88 (-11.41; 23.18) | 6.81 (-12.06; 25.68) | 2.25 (-21.33; 25.82) |
|  |  |  |  |
| **Dichotomized** | *OR (95%CI)* | *OR (95%CI)* | *OR (95%CI)* |
| Daily breakfast | 1.18 (0.22; 6.23) | 1.75 (0.25; 12.37) | 3.49 (0.26; 47.09) |
| ≤ 2 sweet beverages a day | 1.63 (0.87; 3.06) | 1.68 (0.84; 3.35) | **2.43 (1.04; 5.67)*** |
| Outside play ≥ 1 hour a day | 1.63 (0.70; 3.79) | 2.78 (0.96; 8.00) | 2.03 (0.61; 6.80) |
| TV viewing ≤ 2 hour a day | 0.63 (0.32; 1.25) | 0.50 (0.08; 0.44) | 0.62 (0.25; 1.51) |

Note: beta and OR with 95% Confidence Interval (CI) for children in intervention condition, selection based on number of additional sessions, compared to the children in the control condition (n=288). Asterisks indicate significance level: * p<0.05, ** p<0.01, *** p<0.001.
