# Supplementary material for: Promotion of a healthy lifestyle among 5-year-old overweight children: health behavior outcomes of the 'Be active, eat right’ study
Source: BMC Public Health. 2014 Jan 21;14:59. doi: 10.1186/1471-2458-14-59 (PMC3911965; doi:10.1186/1471-2458-14-59)
Supplement: Additional file 4: Table S4 — Results from the regression analyses predicting health behavior outcomes based on the behavior discussed during the well-child visit. [file 1471-2458-14-59-S4.docx]

**Table S4** Evaluation of intervention effects based on the behavior discussed during the well-child visit

|  | Intervention condition (n) | Control condition (n) |  |  |
| --- | --- | --- | --- | --- |
|  | Discussed/ available for analysis | Total/ available for analysis | Odds Ratio (95%CI)¹ | Odds Ratio (95%CI)² |
| Having breakfast (daily) | 60/ 19 | 288/ 133 | 0.59 (0.09;4.06) | 0.59 (0.09; 4.13) |
| Drinking sweet beverages (≤ 2 glasses a day) | 134/ 46 | 288/ 125 | 1.79 (0.89; 3.61) | 1.79 (0.88; 3.63) |
| Playing outside (≥ 1 hour a day) | 104/ 37 | 288/ 121 | 1.18 (0.46; 2.73) | 1.10 (0.44; 2.82) |
| Watching television (≤ 2 hours a day) | 65/ 22 | 288/ 122 | **0.34 (0.13; 0.92)*** | **0.34 (0.13; 0.93)*** |

¹ Odds Ratio (95% Confidence Interval) for the intervention condition vs. control condition (reference) at follow-up from regression model unadjusted for cluster, corrected for time between measurements and age at baseline.

² Odds Ratio (95% Confidence Interval) for the intervention condition vs. control condition (reference) at follow-up from regression model adjusted for cluster, corrected for time between measurements and age at baseline.

Note: **Bold** printed numbers indicate statistically significant behavior change between intervention and control condition, asterisks indicate significance level: * p<0.05, ** p<0.01, *** p<0.001.
